# Allogenic Umbilical Cord Tissue as a Scaffold for Ankle Osteoarthritis

**DOI:** 10.7759/cureus.46572

**Published:** 2023-10-06

**Authors:** Ashim Gupta, Adarsh Aratikatla

**Affiliations:** 1 Regenerative Medicine, Regenerative Orthopaedics, Noida, IND; 2 Regenerative Medicine, Future Biologics, Lawrenceville, USA; 3 Regenerative Medicine, BioIntegrate, Lawrenceville, USA; 4 Orthopaedics, South Texas Orthopaedic Research Institute, Laredo, USA; 5 Medical School, Royal College of Surgeons in Ireland, Dublin, IRL

**Keywords:** wharton’s jelly, umbilical cord, regenerative medicine, osteochondral defect, ankle, talus, ankle osteoarthritis, osteoarthritis

## Abstract

Osteoarthritis (OA) of ankle followed by injury to the talus is one of the most common disorders of ankle. Traditional treatment modalities have limitations and do not address the etiopathogenetic cause of OA. Perinatal tissue-derived biologics such as umbilical cords have shown potential for musculoskeletal regenerative medicine applications. This article qualitatively presents the *in vitro*, pre-clinical, clinical, and ongoing scientific literature exploring the application of umbilical cord tissue in the context of ankle OA. We identified only one clinical study wherein allogenic umbilical cord tissue was applied as a scaffold to the degenerated cartilage in the subtalar synovial joint. Administration of umbilical cord tissue is safe and potentially efficacious in patients with ankle OA. However, more *in vitro*, pre-clinical studies and high-powered, multi-center, non-randomized and randomized controlled trials are warranted to further establish the safety and efficacy of umbilical cord to justify its clinical use in ankle OA patients.

## Introduction and background

The tibiotalar joint is greatly prone to physical injuries resulting in the involvement of the articular surface, varying from osteochondral defects/lesions of the talus to secondary development of osteoarthritis (OA) [1]. An osteochondral defect (OCD) of the talus is an injury to the articular surface of the talus and the underlying bone in the ankle joint, mostly on the posteromedial and anterolateral aspects [2]. OA, on the other hand, is characterized by progressive loss of cartilage, sclerosis of subchondral bone, synovial inflammation, and osteophyte formation [3]. An OCD is known to occur in over 6.5% of ankle sprains [4-6]. In contrast, ankle OA has been known to impact about 1% of the population [7]. The OCD of the talus and its potential for progression to OA of the ankle joint can negatively impact patient’s quality of life, causing pain, swelling, reduced range of motion (ROM), and global ankle instability [8,9].

The non-operative treatment options include activity modification, physical therapy, orthotics, and pharmacological agents such as non-steroidal anti-inflammatory drugs [10]. Patients who fail (up to 50%) to improve with conservative non-operative treatment modalities eventually require more invasive treatment options, including surgery for definitive management [11]. Although the short-term outcomes post-surgery are good, the long-term results are less satisfactory [11]. This can be attributed to the lack of durable structural and biochemical properties in the fibrocartilaginous repaired tissue, required for sustained normal joint function and loading [11]. Thus, there is a need for more effective alternatives for the treatment of OCDs of talus and/or ankle OA.

Lately, there has been a noteworthy increase in the use of biologics including ones derived from perinatal tissues such as human umbilical cord for musculoskeletal regenerative medicine [12]. The primary objective of this study is to document the basic science (in vitro), pre-clinical, and clinical outcomes of umbilical cord tissue and derived mesenchymal stem cells (MSCs) for the treatment of ankle OA. The secondary objective is to record the ongoing clinical trials registered on various trial protocol repositories associated with umbilical cord tissue and derived MSCs for the management of ankle OA.

## Review

Search criteria

**Figure 1:**
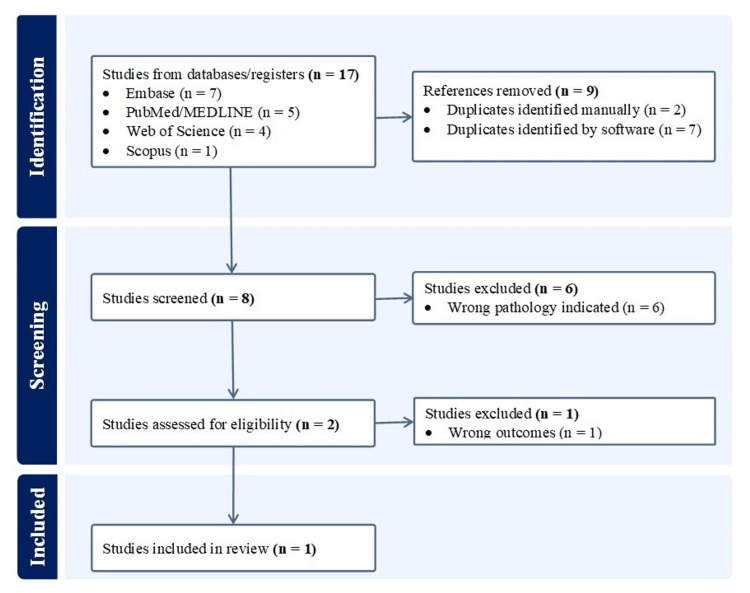
A PRISMA flow diagram outlining the record identification and selection process. PRISMA: Preferred Reporting Items for Systematic Reviews and Meta-Analyses

A search was performed using terms, ('allogenic' OR 'allogeneic' OR 'heterologous' OR 'allograft') AND ('umbilical' OR ‘umbilical cord’ OR ‘umbilical cord blood’ OR 'Whartons jelly' OR 'Wharton jelly') AND (‘talocrural’ OR ‘tibiotalar’ OR ‘subtalar’ OR ‘ankle’ OR ‘tarsus’) AND (‘osteoarthritis’ OR ‘degenerative’ OR ‘osteoarthrosis’ OR ‘arthritis’), in electronic databases including PubMed/MEDLINE, Embase, Scopus, and Web of Science for articles published in English till September 18, 2023. All in vitro, pre-clinical, and clinical studies utilizing umbilical cord tissue and/or derived MSCs for ankle OA were included in this manuscript. Figure 1 illustrates the systematic search performed.

In addition, we searched ClinicalTrials.gov, Chinese Clinical Trial Register (ChiCTR), and Clinical Trials Registry - India (CTRI) using the same search terms to identify registered trials on the use of umbilical cord tissue and/or derived MSCs for the management of ankle OA.

Results

In Vitro Studies

Until now, there have been no published in vitro studies involving the use of allogenic umbilical cord tissue for the treatment of ankle OA.

Pre-clinical studies

Thus far, there are no published preclinical studies involving the use of allogenic umbilical cord tissue for the treatment of ankle OA.

Clinical Studies

A single-center, prospective cohort study assessed whether adjunctive use of umbilical cord tissue/allograft can improve clinical and functional outcomes i.e., reduce post-operative pain and inflammation, improve ROM, and expedite recovery, following arthroscopic repair of the OCD of the talus [11]. In this study, participants diagnosed with an OCD of the talus were recruited. Inclusion criteria included ≥19years old with isolated symptomatic OCD (not secondary to trauma within six months); failed non-operative treatment; minimum dimensions of 0.5cm length, 0.3mm width and maximum dimension of 2.25cm^2^ as confirmed via post-operative computed tomography (CT) scan and intra-operative measurement; and <15^o^ hindfoot valgus, <5^o^ hindfoot varus and stable ankle joint. Exclusion criteria included diabetes with HbA1c >7.5; history of substance abuse; BMI >35Kg/m^2^; currently on radiation or chemotherapy; mentally compromised; or undergoing any concomitant surgery. In eligible participants, after the arthroscopic procedure, a cryopreserved umbilical cord allograft (cut to the size of defect) was placed into the defect. Participants were followed post-operatively at 6, 12, 24, and 52 weeks. The primary outcome measure was Ankle Osteoarthritis Scale (AOS), and secondary outcome measures included, Foot and Ankle Ability Measure (FAAM), Visual Analog Scale (VAS), Short Form-36 (SF-36), and OCD healing as assessed by CT scan/MRI. A total of 10 participants (out of 159 prescreened and 31 screened) were enrolled in this study. All of the participants underwent arthroscopic debridement of the OCD of the talus (three right and seven left ankles) followed by placement of umbilical cord tissue. All outcome measures showed improvement at 52 weeks compared to pre-operative scores. Specifically, the AOS pain and difficulty score, FAAM and SF-36 scores improved from baseline to 52 weeks of follow-up, but this improvement was not statistically significant (p>0.05). On the other hand, VAS score showed significant (p<0.05) improvement at 24 weeks (p=0.043) and 52 weeks (p=0.015) of follow-up compared to baseline. In addition, there was a significant reduction in the size of the OCD and associated bone marrow lesions. No adverse events related to umbilical cord tissue were reported throughout the duration of the study. Despite limitations such as small patient cohort and lack of a control or comparative group, this is one of the first prospective studies demonstrating the safety and potential efficacy of umbilical cord tissue in patients with an OCD of the talus.

Ongoing Clinical Studies

As of September 18, 2023, there are no clinical trials registered on ClinicalTrials.gov, Chinese Clinical Trial Register (ChiCTR), or Clinical Trials Registry - India (CTRI) to study the effects of the umbilical cord tissue for the treatment of ankle OA. 

## Conclusions

In spite of our thorough search of multiple electronic databases, based on our search strategy and pre-defined inclusion/exclusion criteria, only one clinical study fit the scope of our manuscript. In addition, no ongoing clinical trials are registered on any clinical trial protocol registries.

It was observed in this clinical trial that application of umbilical cord tissue as a scaffold to the arthroscopic repair of an osteochondral lesion of the talus resulted in superior functional and clinical outcomes. Despite constraints, the aforementioned study demonstrated that application of cryopreserved umbilical cord tissue in the setting of OCDs is safe and potentially efficacious as a scaffold. However, in vitro and pre-clinical studies along with appropriately powered, multi-center, prospective non-randomized, and randomized controlled studies are warranted to further assess the safety and efficacy of umbilical cord tissue and ultimately justify its clinical use in ankle OA patients.
